# Evaluation Tool Development for Food Literacy Programs

**DOI:** 10.3390/nu10111617

**Published:** 2018-11-02

**Authors:** Andrea Begley, Ellen Paynter, Satvinder S. Dhaliwal

**Affiliations:** School of Public Health, Curtin University, Perth 6152, Australia; ellen.paynter@curtin.edu.au (E.P.); s.dhaliwal@curtin.edu.au (S.S.D.)

**Keywords:** food literacy, questionnaire, validity, internal consistency, evaluation, public health

## Abstract

Food literacy is described as the behaviours involved in planning, purchasing, preparing, and eating food and is critical for achieving healthy dietary intakes. Food literacy programs require valid and reliable evaluation measures. The aim of this paper is to describe the development and validation of a self-administered questionnaire to measure food literacy behaviours targeted by the *Food Sensations^®^ for Adults* program in Western Australia. Validity and reliability tests were applied to questionnaire item development commencing with (a) a deductive approach using Australian empirical evidence on food literacy as a construct along with its components and (b) adapting an extensively-tested food behaviour checklist to generate a pool of items for investigation. Then, an iterative process was applied to develop a specific food literacy behaviour checklist for program evaluation including exploratory factor analysis. Content, face, and construct validity resulted in a 14-item food behaviour checklist. Three factors entitled Plan & Manage, Selection, and Preparation were evident, resulting in Cronbach’s alpha 0.79, 0.76, and 0.81, respectively, indicating good reliability of each of these factors. This research has produced a validated questionnaire, is a useful starting point for other food literacy programs, and has applications globally.

## 1. Introduction

Food literacy is a term used to encompass the knowledge, skills, and behaviours involved in planning, purchasing, preparing, and consuming healthy meals and snacks [1,2,3,4,5]. Evidence suggests poor-quality diets are partly the result of a lack of this knowledge and these skills [6,7]. Thus, food literacy is described as a platform with which to support the development and maintenance of healthy dietary behaviours [1]. Discussion continues in the literature on what needs to be measured including knowledge, attitudes, and behaviours [4,5]. Two approaches are currently being used to produce measures of food literacy using self-reported questionnaires. There are research efforts to develop universal tools using theoretical models tested with general populations [4,5,8], and the second approach is development specifically for program evaluation. This paper will focus on the development of a measurement tool for program evaluation.

Programs that aim to improve food literacy must be evaluated in order to ensure effectiveness; however, systematic reviews of these types of programs generally demonstrate poor evaluation including the lack of use of validated tools, which has changed little between 2014 [9] and 2018 [10]. The gap in development of valid and reliable tools was recognised over 15 years ago, particularly for programs focusing on low-income populations [11,12]. In Australia, there is a deficit of well-evaluated programs [9,13]. Limited evaluation is due to time and resource constraints and challenges with lower literacy levels of the participant group. Evaluation measures need to be designed to achieve a number of respondent burden considerations such as literacy and numeracy, cognitive load, and time to complete in order to avoid detracting from the time available for program delivery [14,15,16]. Validity should be assessed and further modifications made based on feedback from experts and the target group [16,17]. Once a suitable sample has been collected, reliability should also be evaluated [18,19].

Evaluation of programs that aimed to improve the planning, selection, and preparation of foods started in the United States of America (USA) in the 1990s [20,21,22] (Expanded Food and Nutrition Education Program (EFNEP), Food Stamp Education, Cooking with a Chef, and Cooking Matters). First efforts in the United Kingdom (UK) were published in 2011 with Cook Well [23,24] and more recently Australia with the Jamie Oliver Ministry of Food program [25]. The evaluation aim is to pragmatically align the program objectives and content with an evaluation measure. The earliest and most extensively evaluated nutrition education program is the EFNEP run by the Department of Agriculture in the USA since 1969. This program measures food literacy-related behaviour changes (called food resource management and nutrition practices) using a behaviour checklist [19,26,27,28]. The core EFNEP behaviour checklist is a 10-item checklist developed in 1997 and with evaluation determined to be a practical, valid, and reliable measure [28,29]. The extensive testing process including validity and reliability considerations has included validation with serum carotenoids (criterion validity) and 24 h recalls (convergence validity) data [16,19,29] in addition to exploratory factor analysis [28,30]. Through focus groups and other research to assess usability and readability, the questions have been re-worded to elicit the information needed and the format of the questionnaires tested [31,32].

Improving food literacy (food knowledge and skills) is an established strategy utilised by governments to address chronic disease and poor dietary intake in Australia, acknowledged in the 1992 Food and Nutrition Policy. Western Australia (WA) has been proactive in developing practical nutrition education programs, beginning with Food Cent$ developed by the Department of Health in the mid-1980s [33]. Foodbank WA developed the *Food Sensations for Adults* (*FSA*) in 2011 [34], which is based on the key messages of a previous program known as Food Cent$ [33]. This program was reviewed and re-developed in 2015 based on formative research and best practice guidelines developed for the Department of Health [35]. Foodbank WA successfully tendered for a community services request to conduct an adult food literacy program for the Department of Health, WA from March 2016 to June 2018 using *FSA*.

The primary target group for the program are all Western Australian adults from low- to middle-income households with low food literacy who want to increase their food literacy skills. It is a nutrition and cooking program and comprises four weekly, two-and-half-hour sessions covering four core modules (healthy eating, label reading, meal planning, and food preparation) and two out of a possible four optional modules; half of each session is spent cooking and eating together. The Health Belief Model and Social Learning Theory guide *FSA* delivery [36,37]. The program utilises the four constructs (perceived susceptibility, perceived severity, perceived benefits, and perceived barriers) of the Health Belief Model to predict and influence behaviour change and builds self-efficacy by operating as a cue to action, utilising goal setting from the Social Learning Theory. The aim of this paper is to describe the development and validation of a self-administered questionnaire to measure food literacy behaviour for the *FSA* program delivery in WA.

## 2. Materials and Methods

The methods used were adapted from the approaches to scale development outlined by Townsend [12] and DeVellis [38], which start with proposing suitable scale or questionnaire items using a series of validation processes. The questionnaire items are then administered to large, independent samples of subjects in order to determine the latent correlations between the scale items; finally, the best items are selected for a final questionnaire subset of these items. This research started with a deductive approach using Australian empirical evidence on food literacy as a construct along with its components [1], an adapted and extensively tested behaviour checklist of items to generate a pool of items for testing [29,31,39] and then applied an iterative process of developing a food literacy behaviour checklist for program evaluation.

### 2.1. Stage 1 Questionnaire Development

#### 2.1.1. Content Validity

The primary author observed initial program delivery over the first two months (6 programs, 24 sessions) in order to align the curriculum with the questionnaire development. The focus was the impact of the program on self-reported behavior change and self-efficacy. Questionnaire development followed a stepwise process starting with developing a pool of food literacy behaviour questions derived from the program logic model, service outcome objectives, key messages as observed in the program, and written lesson plans, in addition to questions selected from the literature including the EFNEP behaviour checklist core 10 items related to food resource management and food safety. An important consideration in using or adapting questionnaires developed for other programs is to consider how these align with the objectives of the intervention [22]. The four domains of food literacy from empirical research were identified, and questions that best aligned with these domains were selected [1].

Foodbank program facilitators provided content and format feedback during questionnaire development and testing, as they had a key role in the administration of the evaluation tool with participants. Item selection in version 1 was also discussed with four food literacy experts with experience in program delivery or conceptualizing food literacy in Australia to determine the overall emphasis of the measure.

Version 1 of the questionnaire included a food literacy behaviour checklist in addition to questions to assess characteristics of participants. The primary characteristic was income, which was extrapolated from postcode and converted to the Australian Bureau of Statistic’s Socio-Economic Indexes for Areas (SEIFA) decile ranking of the Index of Relative Socio-economic Disadvantage [40]. Deciles 1 to 7 were considered low-to-middle income and 8 to 10 high-income. Other questions assessed gender, age, household structure, education, employment, birth in Australia, and identification as Aboriginal or Torres Strait Islander. Three monitoring questions on responsibility for meals and shopping, and self-assessment of cooking skills, were included from the Department of Health’s (WA) Nutrition Monitoring Surveillance Survey [41].

#### 2.1.2. Face Validity

Face validity assesses whether a tool measures what it purports to measure, and this is a particularly important consideration for low literacy audiences. Methods used assessed acceptability and comprehension of the items in the target population, such as literacy; numeracy of the target groups; and testing of the cognitive load, defined as the amount of mental effort used in working memory. The method used to establish how the target group understood questions included observation and discussion with participants whilst completing questionnaires pre- and post-program to establish face validity [32]. Participants provided feedback on the wording of questions and relevance of some of the food literacy behaviours. Discussions with the Department of Health WA on the service level outcomes led to the addition of four short dietary questions to assess baseline and changes in dietary intake based on state-based surveys [42,43] to produce version 2 of the questionnaire.

#### 2.1.3. Construct Validity

The purpose of this stage was to test the food literacy behaviour questions using participant data using version 2 during a pilot period to examine the performance of each question. Questionnaires were completed by participants attending *FSA* in the third to sixth months of program delivery (*n* = 145) and were used to identify aspects of the questionnaire using the distribution of responses. Questionnaires were revised based on feedback and collected information on participants both on completion of the last session and three months after completion of the program. Three monthly follow-up phone calls were made with pilot participants (*n* = 12) to discuss food literacy behaviour change as a result of the program. The pre-, post-, and follow-up questionnaires were then finalised by assessing plain language requirements and conducting readability assessments. The Flesh Kincaid reading formula was used to test the reading level [44], resulting in pre- and post-program questionnaires that can be viewed in Supplementary Figure S1.

### 2.2. Stage 2 Questionnaire Testing

#### 2.2.1. Factor and Reliability Analysis

Exploratory factor analysis was carried out using data from questionnaires in which participants answered all questions of the food literacy behaviour tool. Thaw meat at room temperature was not included in the analysis for several reasons: (1) the item was added to the questionnaire at a later stage and was often left unanswered in the later version of the questionnaire, so it would reduce the sample size; (2) the item was not found during early data exploration tocluster with any other items or with load sufficiently high as an independent factor. One item (Run out of money for food) was negatively worded and was reverse coded prior to analysis. Adequate sample size was indicated by a Kaiser-Meyer-Olkin value [45]. Bartlett’s test of sphericity was applied to assess if the data was suitable for factor analysis [45]. Internal consistency was assessed using exploratory factor analysis to assess if questionnaire items cluster into one or more groups that have a certain meaning that is related to the program content. Exploratory factor analysis was carried out using three factors for extraction based on guidance from the scree plot; generalised least-squares extraction method to identify the most common factors and varimax rotation as the factors were predicted to be predominantly independent. A factor loading of ≥0.4 was used as the threshold for inclusion of a questionnaire item into a particular factor [46]. Cronbach’s alpha was used to assess reliability of the factors, with α ≥ 0.7 used as a cut-off for an acceptable score [47]. Statistical analysis was conducted using SPSS (IBM) version 25.

#### 2.2.2. Sample

Participants (*n* = 1598) attending 131 *FSA* programs between May 2016 and August 2017 were encouraged to complete the questionnaire before starting the first session. Programs were conducted in metropolitan and regional WA.

### 2.3. Ethics Approval

Ethics approval was obtained from the Human Research Ethics Committee at Curtin Human Research Ethics Committee (RDHS-52-16). Participants were provided with a verbal explanation of the purpose of the research at the start of their first lesson and a written research information sheet. Written consent was obtained prior to questionnaire administration. 

## 3. Results

### 3.1. Stage 1 Food Literacy Behaviour Checklist Development

#### 3.1.1. Content Validity

An original list of previously used 27 behaviour questions drawn from EFNEP [30] along with other research [23] was used as the starting point and narrowed down to 18 behaviour items for the pilot testing for face validity. Consideration was given to colloquial terms, and these were changed as necessary such as ‘grocery’ used in the US to ‘shopping’ in Australia. Items selected were to be consistently answered on the same Likert scale of frequency from ‘never’ (1) to ‘always’ (5). Table 1 demonstrates the alignment of each session’s objectives, key messages, and original behaviour checklist items. Food literacy experts provided feedback on the draft questionnaire questions with respect to format and topics.

#### 3.1.2. Face Validity

Participants completed version 1 of the pre- and post-questionnaires over a two-month period. Feedback from program participants and staff observations informed revisions to the questionnaire. Changes included improving wording and removing questions that were difficult to understand or considered not useful. Three food behaviours were removed as participants commented on the relevance or difficulty in answering, e.g., Waste or throw out food was not something participants considered they did and consider insulting to be asked and Use the plate method to include all food groups was not interpreted to reference the Australian Guide to Healthy Eating. Five other food behaviour items were re-worded to make them clearer, e.g., Make a successful recipe from basic foods was simplified to Try a new recipe. Eat takeaways or fast food outside or at home was removed, as a short dietary question was added to assess frequency of takeaway or fast food consumption. 

Participants also commented on difficulty in applying the five-point scale, being unsure of the difference between ‘seldom’ and ‘sometimes’ in relation to assessing frequency at which they had done the food literacy actions. Behaviour checklist items were reduced to 15 behaviours, and frequency response scale was changed to four frequencies: ‘never’, ‘sometimes’, ‘most of the time’, and ‘always’ in version 2 of the questionnaires. ‘Seldom’ and ‘sometimes’ were coded as one response in existing questionnaires for future analysis.

#### 3.1.3. Construct Validity

Descriptive statistics including score ranges and changes in frequency pre- and post-program were assessed (*n* = 145). A particular focus was assessing which food literacy behaviours were practiced frequently and infrequently at the start of the program. Analysis of the participant data from the administration of version 2 of the pre- and post-questionnaires demonstrated that two behaviours were frequently practiced (>75%) at the start of the program Compare prices to save money and Wash your hands before cooking and one behaviour that was indicated infrequently Run out of money for food. (<15%).

The food literacy behaviour checklist was reduced to 14 items with Compare prices to save money removed, as there was another question asking about prices of healthy foods, and Wash hands before cooking was replaced with Thaw meat at room temperature based on program facilitator’s revision of lesson plan key food safety messages. Run out of money for food was retained, as it provided evidence about the participants attending. The pre- and post-questionnaires were tested for reading level using the Flesh Kincaid reading formula and were found to measure a reading ease score of 78 (the aim for most public documents is to be at 60 or above) [48] and to be at the 5th reading grade level, which is suitable for people of lower literacy [49]. The questionnaires were designed to be self-administered in a group setting in a short time period (approximately 10 min) with supervision by one facilitator. The final versions of the pre- and post-questionnaires were distributed to facilitators for use.

### 3.2. Stage 2 Food Literacy Behaviour Checklist Testing

#### 3.2.1. Response Rate

A total of 1012 respondents (response rate was 63.3%) from 123 programs completed the pre-program questionnaire, or part of the questionnaire. Not all participants consented to be involved in the research or attended the first lesson. An additional eight programs were not able to be evaluated, due to participants’ low literacy and/or mental health or intellectual disability. A final sample of 882 was used for the analysis, in which missing data in participant’s questionnaires was at random and no questions were commonly missed.

#### 3.2.2. Participant Demographics 

Nearly three quarters (71%) of attendees were classified as from low- or middle-income as measured by the Australian Bureau of Statistics Socioeconomic Index of Area (SEIFA) in comparison to 58.5% of the Western Australian population in the 2017 Census of Population and Housing [40] (Table 2). Approximately 82% of participants identified as female. Participants were from a wide range of ages; however, the most represented age group was 26–35 years. The majority of attendees did not currently work; 19% were unemployed, 18% had house-duties, 14% were retired, and 9% were unable to work. Less than half (44%) of participants reported their education level at attending or completing high school, and 52% of participants had completed a certificate/diploma or university degree. Just over half (52%) of the participants were born in Australia, and 7% identified as Aboriginal or Torres Strait Islanders. Data presented in Table 2 includes the Western Australian 2016 Census data [50], which is shown alongside as a comparison. Unfilled space indicates where comparable data was not able to be obtained.

#### 3.2.3. Factor Analysis of Food Literacy Behaviours Tool and Reliability of Factors

**Figure 1:**
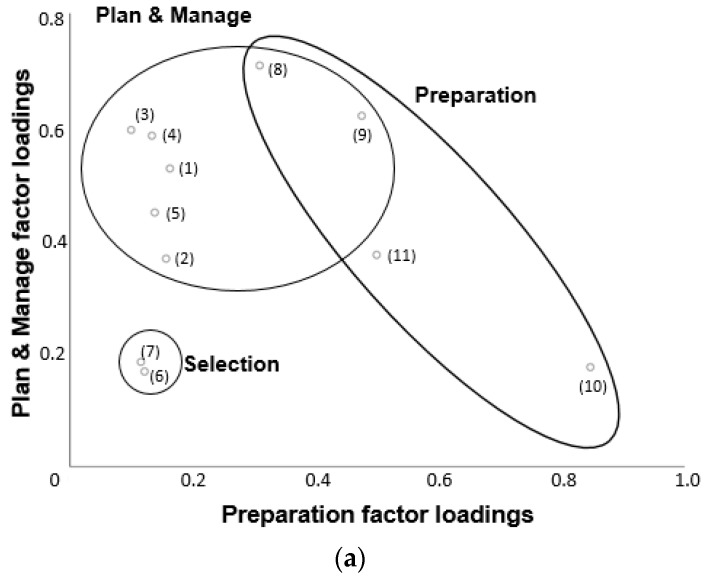

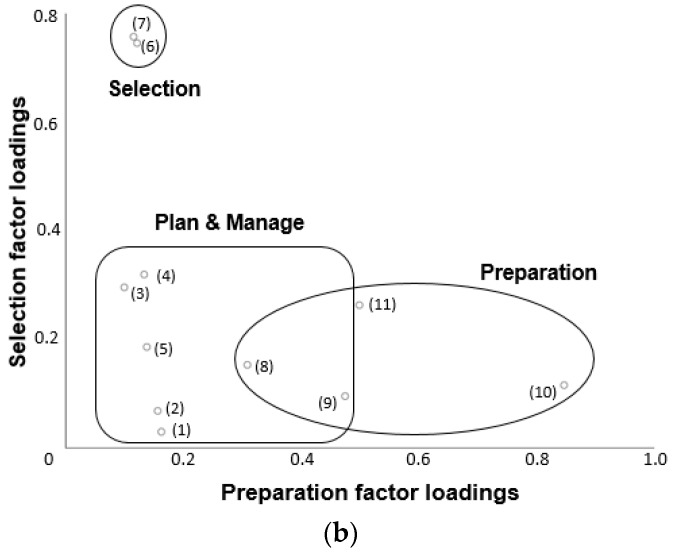
2-Dimensional plots of the three food literacy behaviours factors and the factor loadings of the items (**a**) Plan & Manage and Preparation and (**b**) Selection and Preparation.

A total of 882 participants answered all items included in the factor analysis. Adequate sample size was indicated by a Kaiser-Meyer-Olkin value of 0.859. Bartlett’s test of sphericity (*p* < 0.0001), indicated that the data was suitable for factor analysis. In order to examine the food literacy behaviour checklist in more detail, exploratory factor analysis identified three factors relating to food literacy behaviours that have been labelled Plan & Manage, Selection, and Preparation (Table 3). The clustering of the questionnaire items into these factors can be visualised in Figure 1. Two items, Cook meals at home using healthy ingredients and Feel confident about cooking a variety of healthy meals, loaded on both Plan & Manage and Preparation.

Items that did not meet the specified loading threshold for any factor were Compare the prices of foods to find the best prices on healthy foods and Run out of money for food. Cronbach’s alpha was used to explore internal consistency of each of the defined food literacy behaviour factors, calculated to be 0.79, 0.76, and 0.81 for Plan & Manage, Selection, and Preparation, respectively, indicating good reliability of each of these factors, see Table 3.

## 4. Discussion

This research has demonstrated a systematic approach to developing questionnaire items validated for use in evaluating a food literacy program. This method and its resultant tool have applications for other similar programs globally. In addition to producing a validated food literacy behaviour checklist tool, the internal consistency reliability analysis gives confidence that the questions can detect change and has identified the most useful items for three constructs to be used in further analysis. The refinement of the tool into specific food literacy behaviours as defined factors, Plan & Manage, Selection, and Preparation, can be utilised for a more in-depth analysis. For example, to further investigate the relationship between each food literacy behaviour (e.g., Plan & Manage) with dietary intake and the association with the factors with changes in food literacy behaviours following completion of the program. These three factors correlate with the domains of food literacy from Australian empirical evidence [1]. 

Pragmatically starting with a valid and reliable behaviour checklist tool for measuring change was advantageous for two reasons; firstly, it provides a way to develop a questionnaire in which the funding body requires evaluation to commence shortly after the program delivery has commenced and secondly where there is not sufficient time or resources to develop a questionnaire using extensive research processes [16,29,51]. The strength of the EFNEP behaviour checklist tool for this study is the focus on its ongoing development [52], including regular review to continue to improve evaluation to document effectiveness in changing behaviours in adults [29,31,32,39]. The evaluation tool presented in this paper has been constructed for this program in an Australian context, and this may not be directly applicable for other programs. The only other Australian work has been a content validation process for the survey instrument used in the Jamie Oliver Ministry of Food program, which focuses on cooking confidence and skills [25], and other general research studies often only focus on one aspect of food literacy such as cooking and result in long evaluation tools not always suitable for program evaluation [8]. 

A strength of this evaluation tool is its development with the target population and the sample size. The demographic characteristics of participants indicate they are the target group for this program: three-quarters are from low to middle socioeconomic status postcodes; a high proportion are female; and there is high cultural diversity, with 50% born overseas and a higher proportion of people identifying as Aboriginal or Torres Strait Islander compared to census information [50]. Previous reliability analysis specific to food literacy-type program evaluation has been with small sample sizes [20,23,28]. General tool development studies report development with highly educated people and small sample sizes, for example, Grea Krause et al. (2018) developed a short food literacy questionnaire with 347 people who mostly had tertiary education [4] and others with highly educated Caucasian respondents [8,53]. 

The questions that were not included in the final food literacy behaviour factors were Compare the prices of foods to find the best prices on healthy foods, Run out of money for food, and Thaw meat at room temperature. If a lower factor loading of >0.3 threshold is applied to the first two items [12], they loaded on appropriate factors; Comparing the prices of food to find the best prices on healthy foods loaded strongest on the Selection factor, and Run out of money for food loaded strongest on the Plan & Manage factor. Run out of money for food and Thaw meat at room temperature are single items reflecting food security and food safety, respectively, and one of the considerations when assessing constructs that are not directly measureable is that single item measures are less powerful in this type of analysis [38]. Canadian results have demonstrated that food literacy skills are not correlated with levels of food insecurity [54]. Thaw meat at room temperature was added to the questionnaire as the previous food safety indicator Wash hands before cooking. Handwashing is generally agreed to be a primary focus for food safety education [55], but this behaviour was self-reported frequently for participants at the start of this program. Whilst the 5-point scale is mostly used in EFNEP tools, and numerical scores from 1 to 5 are assigned, some programs have used a four point scale version from never to always, assigning scores from 1 to 4 in which 4 is the highest frequency [4,16].

The testing of the EFNEP behaviour checklist has predominantly demonstrated strong Cronbach’s alpha values; one domain for food resource management such as Planning meals head of time and one for nutrition practices that included Thinking about healthy food choices and Using the food label [39,56]. Hoerr et al. 2011 found exploratory factor analysis on data from 750 EFNEP participants found factor selection from the core 10 behavioural items resulted in one construct: food planning/shopping (0.62) [30]. In comparison, the *FSAs* questionnaire is likely to have more constructs due to the addition of cooking questions. The literature acknowledges that theorising cooking behaviour and measuring food preparation abilities are difficult [8,57]. There is limited program-specific development of preparation and cooking items. The first two studies to report on validity and reliability for cooking specifically were Cook Well in the UK [23] and Cooking with a Chef in the US [20]. Reliability testing found high Cronbach’s alpha values for cooking confidence and knowledge [23] and for techniques, attitudes, and self-efficacy related to fruits and vegetables [20]. Since these publications, confidence related to self-efficacy and the frequency of cooking from basic ingredients has been used in evaluation in other programs [24,58]. Food-safety items have previously been shown to have low Cronbach’s alpha values [28], demonstrating the challenge of developing evaluation tools that align with program objectives and content. 

Any tools used in program evaluation in which participants have literacy and numeracy limitations need to have a low respondent burden and be easily administered in a group setting. Previous experience has suggested two pages as the limit that participants can fill out, with preference given to closed-ended questions [15,23]. It was challenging to word questions to maximise comprehension by participants. The literacy levels of the WA population have been considered in the questionnaire development, as participant education levels are similar to the WA Census of Population and Housing results. The implications for designing evaluation tools are consideration of the literacy, numeracy, and problem-solving ability of the general population in addition to overall respondent burden. The design challenge is demonstrated by the general literacy results for Australia. It has been estimated that the average Australian reading level is at year 8 level, or 13 years of age [59]. Results available in Australia from the 2011–2012 Programme for the International Assessment of Adult Competencies indicate that 45% of the population do not have literacy levels that enable them to understand everyday life written information [60]. Community-based programs rely on voluntary participation, and therefore data collection contains missing records. Primary reasons for missing records are that not all participants give consent, nor are all questionnaires complete even with considerations of cognitive load, literacy, and layout. 

The most important aspect of an evaluation measure is the extent to which it measures what is it intended to measure—in this case, food literacy intentions and behaviours [28]. The process of developing a reliable and valid scale measure of behaviours of interest, particularly when the behaviours are unobservable such as confidence. For program evaluation, there needs to be a focus on the impact measures in developing a tool that can be administered in a short period of time and takes into account respondent burden. Whilst research continues to elucidate the range of food literacy constructs such as environmental, social, and cultural aspects in general measures being developed, program evaluation tools require a specific focus. Unobservable behaviours or constructs must be assessed through indirect means such as self-report; therefore, reliability is important, particularly when behaviours are complex such as in food literacy.

Limitations of this research relate to measurement errors, which are inherent in any evaluation process, and results from affective and cognitive components of participants [61]. The limitations in self-reporting of food literacy behaviours are the similar as for dietary intakes [62] and height and weight [63]. A response bias in rating the frequency of food literacy behaviours has been identified previously with the EFNEP behavior checklist [22]. When answering questions that require self-rating of abilities or skills, respondents will consider individual attributes that occur in different situations and contexts [64]. The food literacy behavior checklist contains mostly concrete behaviours that hopefully minimize this bias; however, respondents in this study may have over- or under-estimated their food literacy behaviour frequency, which may affect the instrument validity. Participants may want to manage their self-perception to appear more favourable, as with social desirability bias [65]. This type of bias results in a tendency to over-report behaviours that are perceived as socially acceptable and under-report those considered socially undesirable [66]. Social desirability bias will lead to inaccuracy, or missing data and contaminant checklist development. To reduce the impact of this type of bias, participants are given instructions that the questions are not a test, that there are no wrong answers and to think about how they usually do things. Despite these limitations, self-report questionnaires continue to be the traditional tool used in program evaluation due to being easy to administer and low-cost. There is some evidence that self-report compares well to direct in-home observations [67]. There are research efforts to establish other ways to measure behaviour change such as use of technology to collect real-time data, but the time and expense are considerations [68].

This research has developed an evaluation tool based on the published food literacy model established in Australia [1]. Whilst efforts continue to develop valid and reliable instruments, all interventions are likely to have different objectives and delivery, meaning that they will require specific questions related to intended outcomes. Valid and reliable measures are needed to ensure appropriate judgement of program effectiveness and funding allocation. Further testing establishing correlations with related constructs/criteria are important for assessing convergent–divergent validity, predictive validity, and other reliability analyses. Further, changes over time can help determine the reliability and stability of food literacy construct. As our knowledge of food literacy and understanding of how people use skills to eat healthier foods improves with empirical research, we will need to continually adapt programs and evaluation tools. 

## Figures and Tables

**Table 1:** Original item selection aligned with session objectives.

Session	Lesson Objectives	Key Program Messages	Food Literacy Behaviour Checklist Items
Basic healthy eating	Be aware of and understand the Australian Dietary Guidelines. Identify foods that belong in the five core food groups and discretionary foods. Explain why eating a variety of foods from each of the core food groups each day is important for good health and wellbeing. Utilise the Australian Guide to Healthy Eating to determine the number of recommended serves of each food group for their age and gender. Set goals to motivate the trial of healthier eating.	Eat a variety of food every day. Drink plenty of water and limit sugar-sweetened beverages. Know your recommended serves from each food group. Eat more vegetables and fruit. Small changes can make a difference.	Plan meals to include all food groups. Do you think about healthy food choices when deciding what to feed your family or household? Use the plate method to include all food groups.
Label reading and food selection	Demonstrate the skills required to read and interpret a food label to compare products based on health and price. Determine if a food product is high or low in a specific nutrient (e.g., fat, sugar, salt, and fibre). Identify the links between foods, nutrients, and chronic disease. Review goals to motivate the trial of healthier eating.	The nutrition information panel is the most accurate information about a product. Drink plenty of water and limit sugar-sweetened beverages.	Use the Nutrition Information Panel to make food. Use other parts of the food label to select healthy foods.
Meal planning and budgeting	Identify money-saving strategies to use when food shopping. Explain the four steps involved in meal planning. Develop a meal plan to effectively plan and manage a household food menu and budget. Determine methods to modify recipes to increase the nutritional value of a meal. Identify ways to substitute ingredients and improvise to assist with meal planning and food budgeting. Identify the links between foods, nutrients, and chronic disease. Review goals to motivate the trial of healthier eating.	Use unit pricing to compare products. Buying foods from the five food groups helps to make your budget go further and improve your health. Meal-planning can save you money and time. Modify your recipes to make them healthier.	Plan meals ahead of time. Making a food plan or list before you go shopping. Plan meals to include all food groups. Compare prices to save money. Compare prices to save money on healthy foods. Feel confident about managing your money to buy healthy food. Waste or throw out food.
Food safety, preparation, and cooking	Identify how to prepare and store food safely. Prepare at least one new recipe. Learn and practice a wide range of basic cooking skills and techniques.	Follow safe food storage, hygiene, and preparation practices to avoid illness. Cooking food at home is healthier, cheaper, and fast to prepare.	Eat takeaways or fast food outside or at home. Cook meals at home using healthy ingredients. Feel confident about cooking a variety of healthy meals. Make a successful recipe from basic foods. Modify recipes to make them healthier. Wash hands before cooking.

**Table 2:** Demographic characteristics of program participants based on questionnaire responses.

Characteristic		Participant %	2016 WA Census %
Socioeconomic Index ^1^ (*n* = 969)			
	Low	39.0	39.6
	Middle	31.7	18.9
	High	29.3	41.2
Gender (*n* = 1007) ^2^			
	Female	81.5	49.9
	Male	18.5	50.1
Age (*n* = 1006)			
	18–25 years	14.6	10.3
	26–35 years	24.1	15.4
	36–45 years	22.3	14.0
	46–55 years	13.6	13.3
	56–65 years	13.0	11.1
	66 and over	12.4	13.0
Household Composition (*n* = 1004)			
	Couple with child/children	45.8	16.9
	Single person	17.5	23.6
	Couple without child/children	16.9	19.6
	Single parent with child/children	8.6	4.0
	Group/supported accommodation	11.1	3.8
Education level (*n* = 994)			
	Bachelor or higher	25.2	20.5
	Diploma/Certificate	30.8	30.6
	Finished high school	24.5	16.0
	Some high school	18.9	23.1
Employment status (*n* = 991)			
	Unemployed	28.5	7.8
	Part-time	24.6	30.0
	Away from work/house-duties/retired	34.9	5.2
	Full-time	11.6	57.0
Born in Australia ^3^ (*n* = 891)		55.2	60.6
Identify as Aboriginal or Torres Strait Islander ^2^ (*n* = 882)		7.1	3.1

^1^ SEIFA derived from postcode (Index of Relative Socio-economic Disadvantage) [40]. ^2^ Gender distribution in our sample reflects that females are more likely to participate in food literacy programs. ^3^ Added in later version of the questionnaire.

**Table 3:** Food literacy behaviours tool, factors, and factor loadings (*n* = 882) *.

How Often Have You Done the Following Actions in the Last Month? (Available Answers: Never, Sometimes, Most of the Time, Always)	Factors and Factor Loadings		
	Plan & Manage (0.790)	Selection (0.756)	Preparation (0.812)
1. Plan meals ahead of time	**0.525**	0.052	0.238
2. Make a list before you go shopping	**0.423**	0.083	0.152
3. Plan meals to include all food groups	**0.484**	0.320	0.277
4. Think about healthy choices when deciding what to eat	**0.463**	0.349	0.300
5. Feel confident about managing money to buy healthy food	**0.512**	0.187	0.187
6. Use Nutrition Information Panel to make food choices	0.129	**0.734**	0.129
7. Use other parts of food label to make food choices	0.133	**0.779**	0.125
8. Cook meals at home using healthy ingredients	**0.515**	0.210	**0.519**
9. Feel confident about cooking a variety of healthy meals	**0.435**	0.124	**0.651**
10. Try a new recipe	0.132	0.115	**0.659**
11. Change recipes to make them healthier	0.183	0.283	**0.642**
12. Compare prices of foods to find the best prices on heathy foods	0.197	0.366	0.181
13. Run out of money for food	0.308	0.062	0.013
14. Thaw meat at room temperature ^1^	-	-	-

^1^ Not included in exploratory factor analysis. * The questionnaire items are listed in the leftmost column. The three columns alongside show the scores of the items for each of the three factors. Those scores in bold text are above the threshold of 0.4 and thus were accepted to be included in the factors and Cronbach’s alpha analyses, indicated in parentheses following each factor title.

## Supplementary Materials

The following are available online at http://www.mdpi.com/2072-6643/10/11/1617/s1, Figure S1: Final versions of the pre- and post-questionnaires.

## References

[1] Vidgen H.A., Gallegos D. (2014). Defining food literacy and its components. Appetite.

[2] Truman E., Lane D., Elliott C. (2017). Defining food literacy: A scoping review. Appetite.

[3] Azevedo Perry E., Thomas H., Samra H.R., Edmonstone S., Davidson L., Faulkner A., Petermann L., Manafò E., Kirkpatrick S.I. (2017). Identifying attributes of food literacy: A scoping review. Public Health Nutr..

[4] Gréa Krause C., Beer-Borst S., Sommerhalder K., Hayoz S., Abel T. (2018). A short food literacy questionnaire (SFLQ) for adults: Findings from a swiss validation study. Appetite.

[5] Palumbo R., Annarumma C., Adinolfi P., Vezzosi S., Troiano E., Catinello G., Manna R. (2017). Crafting and applying a tool to assess food literacy: Findings from a pilot study. Trends Food Sci. Technol..

[6] McGowan L., Pot G.K., Stephen A.M., Lavelle F., Spence M., Raats M., Hollywood L., McDowell D., McCloat A., Mooney E. (2016). The influence of socio-demographic, psychological and knowledge-related variables alongside perceived cooking and food skills abilities in the prediction of diet quality in adults: A nationally representative cross-sectional study. Int. J. Behav. Nutr. Phys. Act..

[7] Garcia A.L., Reardon R., McDonald M., Vargas-Garcia E.J. (2016). Community interventions to improve cooking skills and their effects on confidence and eating behaviour. Curr. Nutr. Rep..

[8] Lahne J., Wolfson J.A., Trubek A. (2017). Development of the cooking and food provisioning action scale (CAFPAS): A new measurement tool for individual cooking practice. Food Qual. Prefer..

[9] Reicks M., Trofholz A.C., Stang J.S., Laska M.N. (2014). Impact of cooking and home food preparation interventions among adults: Outcomes and implications for future programs. J. Nutr. Educ. Behav..

[10] Reicks M., Kocher M., Reeder J. (2018). Impact of cooking and home food preparation interventions among adults: A systematic review (2011–2016). J. Nutr. Educ. Behav..

[11] Contento I.R., Randell J.S., Basch C.E. (2002). Review and analysis of evaluation measures used in nutrition education intervention research. J. Nutr. Educ. Behav..

[12] Townsend M.S. (2006). Evaluating food stamp nutrition education: Process for development and validation of evaluation measures. J. Nutr. Educ. Behav..

[13] Begley A., Gallegos D., Vidgen H. (2017). Effectiveness of australian cooking skill interventions. Br. Food J..

[14] Townsend M.S., Sylva K., Martin A., Metz D., Wooten-Swanson P. (2008). Improving readability of an evaluation tool for low-income clients using visual information processing theories. J. Nutr. Educ. Behav..

[15] Banna J.C., Vera Becerra L.E., Kaiser L.L., Townsend M.S. (2010). Using qualitative methods to improve questionnaires for spanish speakers: Assessing face validity of a food behavior checklist. J. Am. Diet. Assoc..

[16] Murphy S.P., Kaiser L.L., Townsend M.S., Allen L.H. (2001). Evaluation of validity of items for a food behavior checklist. J. Am. Diet. Assoc..

[17] Kristal A.R., Abrams B.F., Thornquist M.D., Disogra L., Croyle R.T., Shattuck A.L., Henry H.J. (1990). Development and validation of a food use checklist for evaluation of community nutrition interventions. Am. J. Public Health.

[18] Branscum P., Sharma M., Kaye G., Succop P. (2010). An evaluation of the validity and reliability of a food behavior checklist modified for children. J. Nutr. Educ. Behav..

[19] Blackburn M.L., Townsend M.S., Kaiser L.L., Martin A.C., West E.A., Turner B.J., Joy A.B. (2006). Food behavior checklist effectively evaluates nutrition education. Calif. Agric..

[20] Condrasky M.D., Williams J.E., Catalano P.M., Griffin S.F. (2011). Development of psychosocial scales for evaluating the impact of a culinary nutrition education program on cooking and healthful eating. J. Nutr. Educ. Behav..

[21] Pinard C.A., Uvena L.M., Quam J.B., Smith T.M., Yaroch A.L. (2015). Development and testing of a revised cooking matters for adults survey. Am. J. Health Behav..

[22] Auld G., Baker S., McGirr K., Osborn K.S., Skaff P. (2017). Confirming the reliability and validity of others’ evaluation tools before adopting for your programs. J. Nutr. Educ. Behav..

[23] Barton K.L., Wrieden W.L., Anderson A.S. (2011). Validity and reliability of a short questionnaire for assessing the impact of cooking skills interventions. J. Hum. Nutr. Diet..

[24] Hutchinson J., Watt J.F., Strachan E.K., Cade J.E. (2016). Evaluation of the effectiveness of the ministry of food cooking programme on self-reported food consumption and confidence with cooking. Public Health Nutr..

[25] Flego A., Herbert J., Gibbs L., Swinburn B., Keating C., Waters E., Moodie M. (2013). Methods for the evaluation of the jamie oliver ministry of food program, australia. BMC Public Health.

[26] Schuster E. (1988). EFNEP (expanded food and nutrition program). Am. J. Public Health.

[27] Dollahite J., Olson C., Scott-Pierce M. (2009). The impact of nutrition education on food insecurity among low-income participants in EFNEP. Fam. Consum. Sci. Res. J..

[28] Bradford T., Serrano E.L., Cox R.H., Lambur M. (2010). Development and testing of a nutrition, food safety, and physical activity checklist for EFNEP and FSNE adult programs. J. Nutr. Educ. Behav..

[29] Townsend M.S., Kaiser L.L., Allen L.H., Joy A.B., Murphy S.P. (2003). Selecting items for a food behavior checklist for a limited-resource audience. J. Nutr. Educ. Behav..

[30] Hoerr S., Abdulkadri A., Miller S., Waltersdorf C., LaShore M., Martin K., Newkirk C. (2011). Improving measurement of the EFNEP outcomes using factor analysis of the behavior checklist. J. Ext..

[31] Anliker J., Willis W., Montogomery S. The Development and Testing of the Behavioural Checklist Questions for the Efnep Evaluation/Reporting System. https://nifa.usda.gov/sites/default/files/resource/Development%20and%20Testing%20of%20the%20Behavior%20Checklist%20Questions.pdf.

[32] Townsend M.S., Ganthavorn C., Neelon M., Donohue S., Johns M.C. (2014). Improving the quality of data from efnep participants with low literacy skills: A participant-driven model. J. Nutr. Educ. Behav..

[33] Foley R., Pollard C. (1998). Food cent$—Implementing and evaluating a nutrition education project focusing on value for money. Aust. N. Z. J. Public Health.

[34] Butcher L., Chester M.R., Aberle L.M., Bobongie V.J.-A., Davies C., Godrich S.L., Milligan R.A.K., Tartaglia J., Thorne L.M., Begley A. (2014). Foodbank of western australia’s healthy food for all. Br. Food J..

[35] Begley A., Coelho G., Brooks N. (2015). Adult Food Literacy: Best Practice Criteria.

[36] Stokols D. (1996). Translating social ecological theory into guidelines for community health promotion. Am. J. Health Promot. AJHP.

[37] Coleman M.T., Pasternak R.H. (2012). Effective strategies for behavior change. Prim. Care.

[38] DeVillis R. (2012). Scale Development Theory and Applications.

[39] Phelps J., Brite-Lane A., Crook T., Hakkak R., Fuller S. (2017). Demonstrating impact through replicable analysis: Implications of an evaluation of arkansas’s expanded food and nutrition education program. J. Ext..

[40] Australian Bureau of Statistics Census of Population and Housing: Socio-Economic Indexes for Areas (SEIFA). https://researchdata.ands.org.au/census-population-housing-seifa-sa2/643007?source=suggested_datasets.

[41] Pollard C., Harray A., Daly A., Kerr D. Nutrition Monitoring Survey Series 2012 Key Findings. http://ww2.health.wa.gov.au/~/media/Files/Corporate/Reports%20and%20publications/Population%20surveys/13032-nutrition-monitoring-survey-series-2012.ashx.

[42] Tomlin S., Joyce S. Health and Wellbeing of Adults in Western Australia 2012, Overview and Trends. https://ww2.health.wa.gov.au/~/media/Files/Corporate/Reports%20and%20publications/Population%20surveys/2012-HWSS_Adult_Overview_and_Trends_2012.pdf.

[43] Tomlin S., Joyce S., Radomilijac A. Health and Wellbeing of Adults in Western Australia 2015, Overview and Trends. https://ww2.health.wa.gov.au/~/media/Files/Corporate/Reports%20and%20publications/Population%20surveys/Health-and-Wellbeing-of-Adults-in-Western-Australia-2015-Overview-and-Trends.pdf.

[44] Friedman D.B., Hoffman-Goetz L. (2006). A systematic review of readability and comprehension instruments used for print and web-based cancer information. Health Educ. Behav..

[45] Williams B., Onsman A., Brown T. (2010). Exploratory factor analysis: A five-step guide for novices. Aust. J. Paramed..

[46] Stevens J. (2009). Applied Multivariate Statistics for the Social Sciences.

[47] Gliem J.A., Gliem R.R. Calculating, Interpreting, and Reporting Cronbach’s Alpha Reliability Coefficient for Likert-Type Scales. https://scholarworks.iupui.edu/handle/1805/344.

[48] Wilson M. (2009). Readability and patient education materials used for low-income populations. Clin. Nurse Spec. CNS.

[49] Fisher E. (1999). Low literacy levels in adults: Implications for patient education. J. Contin. Educ. Nurs..

[50] Australian Bureau of Statistics Census of Population and Housing, Australia 2016. http://www.abs.gov.au/websitedbs/censushome.nsf/home/2016.

[51] Rees R., Hinds K., Dickson K., O’Mara-Eves A., Thomas J. Communities That Cook: A Systematic Review of the Effectiveness and Appropriateness of Interventions to Introduce Adults to Home Cooking. https://eppi.ioe.ac.uk/cms/Default.aspx?tabid=3322.

[52] Murray E.K., Auld G., Baker S.S., Barale K., Franck K., Khan T., Palmer-Keenan D., Walsh J. (2017). Methodology for developing a new EFNEP food and physical activity behaviors questionnaire. J. Nutr. Educ. Behav..

[53] Carbonneau E., Bradette-Laplante M., Lamarche B., Provencher V., Begin C., Robitaille J., Desroches S., Vohl M.C., Corneau L., Lemieux S. (2017). Development and validation of the food liking questionnaire in a french-canadian population. Nutrients.

[54] Huisken A., Orr S.K., Tarasuk V. (2017). Adults’ food skills and use of gardens are not associated with household food insecurity in canada. Can. J. Public Health.

[55] Medeiros L.C., Hillers V.N., Kendall P.A., Mason A. (2001). Food safety education: What should we be teaching to consumers?. J. Nutr. Educ..

[56] Wardlaw M., Baker S. (2012). Long-term evaluation of EFNEP and snap-ed. Forum Fam. Consum. Issues (FFCI).

[57] Short F. (2003). Domestic cooking skills—What are they?. HEIA J..

[58] Garcia A., Reardon R., Hammond E., Parrett A., Gebbie-Diben A. (2017). Evaluation of the “eat better feel better” cooking programme to tackle barriers to healthy eating. Int. J. Environ. Res. Public Health.

[59] Perkins L., Cohen J. (2008). Meeting patient needs in the hospital setting—Are written nutrition education resources too hard to understand?. Nutr. Diet..

[60] (ABS), Australian Bureau of Statistics Programme for the International Assessment of Adult Competencies, Australia, 2011–12. http://abs.gov.au/ausstats/abs@.nsf/Lookup/4228.0Explanatory+Notes12011-12.

[61] Clark L.A., Watson D. (1995). Constructing validity: Basic issues in objective scale development. Psychol. Assess..

[62] Subar A.F., Freedman L.S., Tooze J.A., Kirkpatrick S.I., Boushey C., Neuhouser M.L., Thompson F.E., Potischman N., Guenther P.M., Tarasuk V. (2015). Addressing current criticism regarding the value of self-report dietary data. J. Nutr..

[63] Dhaliwal S.S., Howat P., Bejoy T., Welborn T.A. (2010). Self-reported weight and height for evaluating obesity control programs. Am. J. Health Behav..

[64] Braun E., Woodley A., Richardson J., Leidner B. (2012). Self-rated competences qusestionnaires from a design perspective. Educ. Res. Rev..

[65] King M.F., Bruner G.C. (2000). Social desirability bias: A neglected aspect of validity testing. Psychol. Mark..

[66] Slater J.J., Mudryj A.N. (2016). Self-perceived eating habits and food skills of canadians. J. Nutr. Educ. Behav..

[67] Bongoni R., Verkerk R., Dekker M., Steenbekkers B. (2015). Evaluation of research methods to study domestic food preparation. Br. Food J..

[68] Hand R.K., Perzynski A.T. (2016). Ecologic momentary assessment: Perspectives on applications and opportunities in research and practice regarding nutrition behaviors. J. Nutr. Educ. Behav..

