# Supplementary material for: Evaluation Tool Development for Food Literacy Programs
Source: Nutrients. 2018 Nov 2;10(11):1617. doi: 10.3390/nu10111617 (PMC6267114; doi:10.3390/nu10111617)
Supplement: Supplementary file 1 [file nutrients-10-01617-s001.pdf]

Food Sensations Start of Program Questions   Initials: ___________

These questions ask about how you plan and prepare food for you and/or your family.

This is not a test! There are no wrong answers. Think about how you usually do things.

1. How often have you done the following actions in the last month? Please put a tick or circle in the box that is the best answer for each question.

Plan meals ahead of time?	Never	Sometimes	Most of the time	Always
Make a list before you go shopping?	Never	Sometimes	Most of the time	Always
Plan meals to include all food groups?	Never	Sometimes	Most of the time	Always
Think about healthy food choices when deciding what to eat?	Never	Sometimes	Most of the time	Always

Run out of money for food?	Never	Sometimes	Most of the time	Always
Feel confident about managing your money to buy healthy food?	Never	Sometimes	Most of the time	Always

Use a nutrition information panel to make food choices?	Never	Sometimes	Most of the time	Always
Use other parts of a food label to make food choices?	Never	Sometimes	Most of the time	Always
Compare prices of foods to find best prices on healthy foods?	Never	Sometimes	Most of the time	Always

Cook meals at home using healthy ingredients?	Never	Sometimes	Most of the time	Always
Feel confident about cooking a variety of healthy meals?	Never	Sometimes	Most of the time	Always
Try a new recipe?	Never	Sometimes	Most of the time	Always
Change recipes to make them healthier?	Never	Sometimes	Most of the time	Always
Thaw meat at room temperature?	Never	Sometimes	Most of the time	Always

2. What do you think of the following statements?

Healthy foods cost more than unhealthy foods?	Strongly Disagree	Disagree	Not Sure	Agree	Strongly Agree
Food has an impact on future health and wellbeing?	Strongly Disagree	Disagree	Not Sure	Agree	Strongly Agree

3. Do you have responsibility for <u>choosing and preparing the household meals</u>?

- Yes all the responsibility ☐ 1
- Yes but share the responsibility ☐ 2
- No responsibility ☐ 3

4. Do you have responsibility for doing the <u>household food shopping</u>?

- Yes all the responsibility ☐ 1
- Yes but share the responsibility ☐ 2
- No responsibility ☐ 3

5. Which of the following best describes your <u>cooking skills</u>?

- Can cook almost anything ☐ 1
- Can cook a wide variety of meals ☐ 2
- Can cook basic meat and 3 vegetables ☐ 3
- Can do basic heating food, use barbeque, boil egg ☐ 4
- Can’t cook/Don’t cook ☐ 5

6. How many <u>serves</u> of fruit do you usually eat <u>each day</u>?

1 serve of fruit is equal to 1 medium piece or 1 cup diced or canned fruit

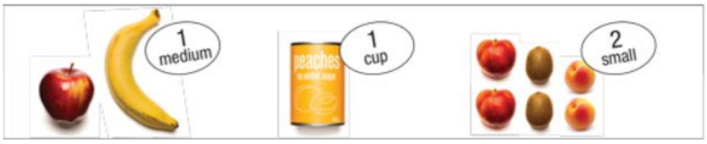

**no serves**	**½ serve**	**1 serve**	**1 ½ serves**	**2 serves**	**2 ½ serves**	**3 serves**	**3 ½ serves**	**4 serves**	**4 ½ serves**	**5 or more serves**

7. How many <u>serves</u> of vegetables do you usually eat <u>each day</u>?

1 serve of vegetables is equal to
1/2 cup of cooked vegetables or 1 cup of salad

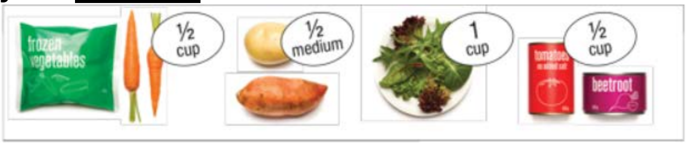

**no serves**	**½ serve**	**1 serve**	**1 ½ serves**	**2 serves**	**2 ½ serves**	**3 serves**	**3 ½ serves**	**4 serves**	**4 ½ serves**	**5 or more serves**

8. How many <u>times a week on average</u> do you eat fast food meals, such as burgers, pizza, chicken or chips from fast food outlets?

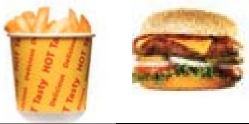

Never/rarely	Less than once a week	1 or 2 times a week	3 or 4 times a week	5 or more times a week

9. How many <u>times a week on average</u> do you drink regular soft drink (not diet), energy drinks, sports drinks, flavoured mineral water or vitamin water?

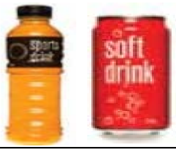

Never/rarely	Less than once a week	1 or 2 times a week	3 or 4 times a week	5 or more times a week

10. Why did you come to this program today? (tick as many options as you need)

Learn about healthy eating and nutrition ☐ 1
Learn to cook or improve confidence with cooking skills ☐ 2
Make healthier meals ☐ 3
Get new ideas for cooking ☐ 4
Learn to read food labels ☐ 5
Improve food budgeting ☐ 6
Make healthier snacks and lunchboxes for children ☐ 7
Other reason/s ______________________________________ ☐

11. These questions help us describe who is attending *Food Sensations.*

Are you? Male ☐ 1 Female ☐ 2

How old are you?
18-25 ☐ 1 26-35 ☐ 2 36-45 ☐ 3 46-55 ☐ 4 56-65 ☐ 5 66 and over ☐ 6

What is your postcode?______________________

Who lives in your house?
Live alone ☐ 1 Live with partner, no children ☐ 2 Single parent living with children ☐ 3
Live with partner and children ☐ 4 Shared house ☐ 5 Supported accommodation ☐ 6
Extended family ☐ 7 other ____________

What is the highest level of education you have completed?
Primary or some high school ☐ 1 Finished high school (leaving) ☐ 2 Trade/apprenticeship ☐ 3
Certificate or diploma ☐ 4 Bachelor degree or higher ☐ 5 other __________________

What is your employment status?
Full-time ☐ 1 Part-time ☐ 2 Casual ☐ 3 Unemployed ☐ 4 Unable to work ☐ 5
Household duties ☐ 6 Retired ☐ 7 Volunteer ☐ 8 Other ______________________________

Where you born in Australia?
Yes ☐ 1 No ☐ 2

Do you identify as Aboriginal or Torres Strait Islander?
Yes ☐ 1 No ☐ 2

Please check you have answered all the questions!

Thank you for completing this questionnaire and please return to the Foodbank WA staff member.

Food Sensations **End of Program Questions** **Initials: ___________**

These questions ask about how you plan and prepare food for you and/or your family.

This is not a test! There are no wrong answers. Think about how you usually do things.

1. How often have you done the following actions in <u>the last month</u>? Please put a tick or circle in the box that is the best answer for each question.

Plan meals ahead of time?	Never	Sometimes	Most of the time	Always
Make a list before you go shopping?	Never	Sometimes	Most of the time	Always
Plan meals to include all food groups?	Never	Sometimes	Most of the time	Always
Think about healthy food choices when deciding what to eat?	Never	Sometimes	Most of the time	Always

Run out of money for food?	Never	Sometimes	Most of the time	Always
Feel confident about managing your money to buy healthy food?	Never	Sometimes	Most of the time	Always

Use a nutrition information panel to make food choices?	Never	Sometimes	Most of the time	Always
Use other parts of a food label to make food choices?	Never	Sometimes	Most of the time	Always
Compare prices of foods to find best prices on healthy foods?	Never	Sometimes	Most of the time	Always

Cook meals at home using healthy ingredients?	Never	Sometimes	Most of the time	Always
Feel confident about cooking a variety of healthy meals?	Never	Sometimes	Most of the time	Always
Try a new recipe?	Never	Sometimes	Most of the time	Always
Change recipes to make them healthier?	Never	Sometimes	Most of the time	Always
Thaw meat at room temperature?	Never	Sometimes	Most of the time	Always

2. What do you think of the following statements?

Healthy foods cost more than unhealthy foods?	Strongly Disagree	Disagree	Not Sure	Agree	Strongly Agree
Food has an impact on future health and wellbeing?	Strongly Disagree	Disagree	Not Sure	Agree	Strongly Agree

3. What were the goals you set yourself at the beginning of the program?

4. What changes have you made based on your goals?

5. How many <u>serves</u> of fruit do you usually eat <u>each day</u>?

1 serve of fruit is equal to 1 medium piece or 1 cup diced or canned fruit

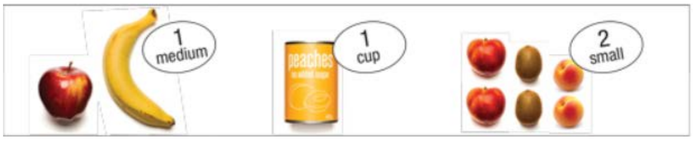

**no serves**	**½ serve**	**1 serve**	**1 ½ serves**	**2 serves**	**2 ½ serves**	**3 serves**	**3 ½ serves**	**4 serves**	**4 ½ serves**	**5 or more serves**

6. How many <u>serves</u> of vegetables do you usually eat <u>each day</u>?

1 serve of vegetables is equal to
1/2 cup of cooked vegetables or 1 cup of salad

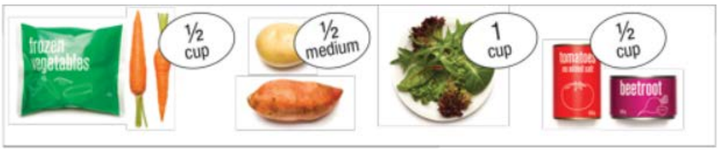

**no serves**	**½ serve**	**1 serve**	**1 ½ serves**	**2 serves**	**2 ½ serves**	**3 serves**	**3 ½ serves**	**4 serves**	**4 ½ serves**	**5 or more serves**

7. How many <u>times a week on average</u> do you eat fast food meals, such as burgers, pizza, chicken or chips from fast food outlets?

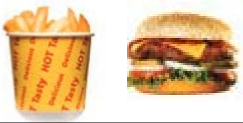

Never/Rarely	Less than once a week	1 or times a week	3 or 4 times a week	5 or more times a week

8. How many <u>times a week on average</u> do you drink regular soft drink (not diet), energy drinks, sports drinks, flavoured mineral water or vitamin water?

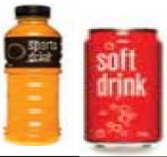

Never/Rarely	Less than once a week	1 or 2 times a week	3 or 4 times a week	5 or more times a week

9. Have you shared any of the program materials with family or friends or others?

Yes ☐ 1 No ☐ 2 Not yet ☐ 3

10. What have you liked most about the Food Sensations program?

____

____

11. Do you have any suggestions for improvement of the Food Sensations program?

____

____

We would like to contact you in 3 months' time to ask about your experiences with Food Sensations. This survey will only take 5- 10 minutes.

You can enter into a draw to win a $200 voucher if you complete all questionnaires. This draw will happen every three months.

Please provide your name and contact details.

Name: ____

Phone Number:____

Email: ____

Or prefer to be sent a paper survey with replied paid envelope

Mailing address: ____

____

All details provided are confidential

Please check you have answered all questions!

Thank you for completing this survey and please return to the Foodbank WA staff member.
